# Anti-Photoaging Activity of *Scutellaria barbata* D. Don (Family Lamiaceae) on Ultraviolet B-Irradiated NIH-3T3 Skin Fibroblast and SKH-1 Hairless Mouse

**DOI:** 10.3390/molecules27123803

**Published:** 2022-06-13

**Authors:** Jong Min Jung, Jong Kyu Choi, Oh Yun Kwon, Seung Ho Lee

**Affiliations:** Department of Nano-Bioengineering, Incheon National University, 119 Academy-ro, Yeonsu-gu, Incheon 22012, Korea; ljjm9659@gmail.com (J.M.J.); carmonster@naver.com (J.K.C.); ohyun1220@naver.com (O.Y.K.)

**Keywords:** *Scutellaria barbata* D. Don, photoaging, UVB, AKT, wrinkle formation

## Abstract

We investigated whether *Scutellaria barbata* D. Don (Family Lamiaceae) (SBD), a traditional medicine used for heat clearing and detoxification, possesses antiphotoaging properties. Pretreatment of NIH-3T3 skin fibroblast cells with non-toxicological levels of water extract of SBD (WESBD) and ethanol extract of SBD (EESBD) restored the expression of procollagen type-1 (*COL1A1*), matrix metalloproteinase-1a (*MMP-1a*), interleukin-6 (*IL-6*), interleukin-8 (*IL-8*), and monocyte chemotactic protein-3 (*MCP-3*) genes following abnormal expression induced by ultraviolet B (UVB) irradiation. WESBD/EESBD administration to the dorsal skin area of hairless mice significantly (*p* < 0.05) inhibited UVB-induced wrinkle formation and epidermal thickness. The WESBD and EESBD treatments also restored the dermal collagen content, which was decreased by the UVB treatment, and normal *COL1A1* and *MMP-1a* expression. Interestingly, both the WESBD and EESBD pretreatments significantly attenuated UVB-induced phosphorylation of protein kinase B (AKT) but not that of mitogen-activated protein kinases (MAPKs). This finding indicates that the antiphotoaging effects of WESBD and EESBD may be related to attenuation of UVB-induced overactivation of AKT phosphorylation. High performance liquid chromatography (HPLC) and mass spectrometry analysis revealed that isorhamentin and scutebarbatine I were major single components of EESBD. These results suggest that WESBD and EESBD may have potential in development as antiphotoaging agents.

## 1. Introduction

The rise in the median age of populations in many countries has focused much attention on skin aging not only for esthetic but also medical reasons. Continuous or repeated exposure to high wavelength ultraviolet (UV) radiation, such as UVB and UVA, for a long time (month or years) is a major cause of photoaging, which is characterized by the formation of wrinkles [1], increased skin thickness, dyspigmentation [2], reduced elasticity [3], and loss of subcutaneous fat [3]. The association of chronic UVB exposure with dermal collagen loss and photoaging is well known. Many antiphotoaging agents (synthetic and natural) aimed at upregulating or downregulating collagen biosynthesis have been developed [4,5,6]. Inflammatory responses are often detected in UVB-exposed skin. Such responses include upregulation of the expression of proinflammatory cytokines, such as interleukin (IL)-6, IL-8, and monocyte chemotactic protein-3 (MCP-3), which are considered a cause of erythema and acute edema in UVB-irradiated skin [7]. An increase in IL-6, IL-8, and MCP-3 is thought to be responsible for a reduction in subcutaneous fat in UVB-irradiated skin [3]. Therefore, proinflammatory cytokines could be a target in efforts aimed at developing antiphotoaging agents.

Mitogen-activated protein kinases (MAPKs) and AKT are well-known intracellular signaling molecules that control various cellular events, including inflammation, apoptosis, and cell proliferation [8,9,10]. The progression of photoaging is associated with abnormal phosphorylation of MAPKs and Akt in UVB-irradiated skin. The latter is thought to be closely related to abnormal expression of photoaging-related genes, such as of procollagen type-1 (*COL1A1*) and matrix metalloproteinase-1a (*MMP-1a*), mediated by UVB [11,12,13]. Therefore, attenuating abnormal phosphorylation of MAPKs and AKT induced by UVB could be an effective strategy for developing antiphotoaging agents.

*S. barbata* D. Don (SBD) is a perennial herb generally reaching up to 35–50 cm in height. SBD consists of branched and quadrilateral stems with toothed leaves. The flowers of *S. barbata* bloom between May and October and it has purple calyx, purple-blue corolla, and two lobed stigma. *S. barbata* is known to grow in paddy fields or wet grasslands. *S. barbata* is a herb used as a traditional medicine in east Asia, including Korea and China. SBD is known as “Banjiryun” in Korea and picked during blooming period (from May to October). Hot water extracts of whole body of SBD have been consumed orally as a decoction to treat inflammation, hemoptysis, stomach pain, and early-stage cancer [14]. Recently, SBD has been used for many prescriptions of herb combination such as Kang Ai Ping Wa and Gong Liu Ning Pian in China [14]. Recent research has revealed that SBD has various biological activities (i.e., antimicrobial, anticancer, and anti-inflammation) [15,16,17]. The potential antiphotoaging effects of SBD have not been elucidated. Therefore, in this study, we investigated the effects of water extract of SBD (WESBD) and ethanol extract of SBD (EESBD) on attenuation photoaging progression induced by UVB irradiation and its underlying molecular mechanisms.

## 2. Results

### 2.1. Non-Toxicological Levels of WESBD and EESBD Restored the Expression of Photoaging-Related Genes

**Figure 1:**
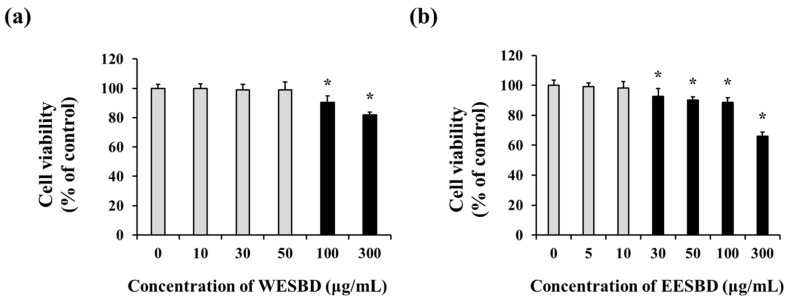
Cytotoxicity assay of water extract of *S. barbata* D. Don (WESBD) and ethanol extract of SBD (EESBD) in NIH-3T3 skin fibroblast cells. Various concentrations of WESBD (0–300 µg/mL) (**a**) and EESBD (0–300 µg/mL) (**b**) were added to NIH-3T3 cells cultured in a 96-well plate and then incubated for 24 h at 37 °C in a CO_2_ incubator. The experiments were repeated three times, with similar results. A representative result is shown. Data are presented as the mean ± SD. *: Indicates a significant difference (*p* < 0.05) compared with the control (no treatment).

To examine the efficacy of SBD in attenuating the photoaging process, SBD was extracted using two different solvents (water and ethanol), and two kinds of SBD-based agents (water extract of SBD (WESBD) and ethanol extract of SBD (EESBD)) were produced. As shown in Figure 1, the addition of 100 µg/mL of WESBD and 30 µg/mL of EESBD to the NIH-3T3 cells induced cytotoxicity. Thus, 25–50 µg/mL of WESBD and 5–10 µg/mL of EESBD were used in subsequent experiments to assess the antiphotoaging efficacy of SBD in vitro.

**Figure 2:**
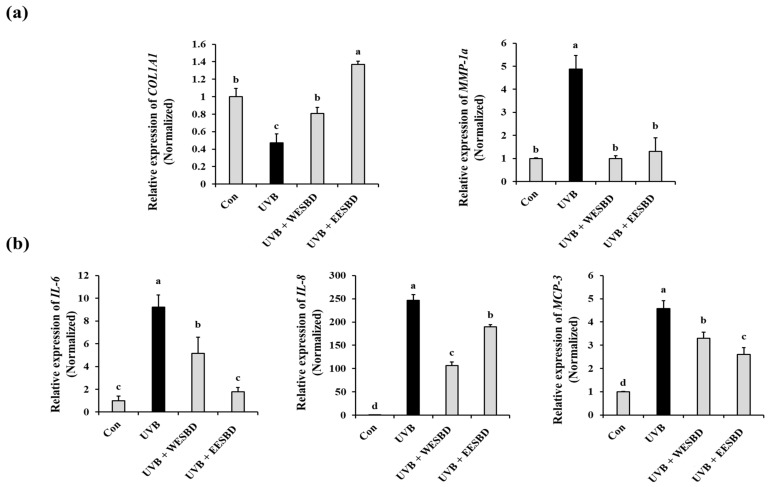
Effect of water extract of *S. barbata* D. Don (WESBD) and ethanol extract of SBD (EESBD) treatments on the expression of photoaging-related gene expression induced by ultraviolet B (UVB) irradiation. Pretreatment with WESBD (50 µg/mL) or EESBD (10 µg/mL) eliminated abnormal expression of type-1 procollagen (COL1A1) (**a**), metalloproteinase-1a (MMP-1a) (**a**), IL-6 (**b**), IL-8 (**b**), and MCP-3 (**b**) induced by UVB irradiation in NIH-3T3 cells. The expression of these genes was determined by quantitative real-time PCR (qRT-PCR) and normalized to that of the glyceraldehyde 3-phosphate dehydrogenase (*GAPDH*) gene. The experiments were repeated three times, with similar results. A representative result is shown. Data are presented as the mean ± SD. Different letters indicate a significant difference between groups (*p* < 0.05).

As skin collagen content is an important indicator of photoaging, we then determined the effects of WESBD and EESBD on the expression of *COL1A1* and *MMP-1a* genes, which are closely linked to collagen synthesis and degradation, respectively. As shown in Figure 2a, *COL1A1* expression was decreased in the UVB-irradiated group as compared with that in the control group and restored in the WESBD- and EESBD-treated groups (*p* < 0.05). In addition, the EESBD treatment (10 µg/mL and 50 µg/mL) significantly attenuated UVB-induced overexpression of *MMP-1a* in NIH-3T3 cells (*p* < 0.05). These results suggest that non-toxicological levels of WESBD and EESBD may have potential in reducing UVB-induced loss of skin collagen content by regulating the expression of *COL1A1* and *MMP-1a*.

### 2.2. Non-Toxicological Levels of WESBD and EESBD Restored the Expression of Photoaging-Related Genes

As repeat UVB exposure can induce inflammation and skin damage, attenuation of UVB-induced inflammatory responses has been recognized as an important way to maintain skin health. Proinflammatory cytokines, such as IL-6, IL-8, and MCP-3 are known to be key regulators of subcutaneous fat loss induced by UVB irradiation. Therefore, these cytokines are primary targets in the development of antiphotoaging agents. In this study, the expression of proinflammatory cytokines (IL-6, IL-8, and MCP-3) was upregulated in the UVB-irradiated NIH-3T3 cells and downregulated in the cells pretreated with non-toxicological levels of WESBD or EESBD (Figure 2b) (*p* < 0.05). These results suggest that both WESBD and EESBD may have potential as antiphotoaging agents by suppressing the UVB-induced increase in proinflammatory cytokines (IL-6, IL-8, and MCP-3).

### 2.3. WESBD and EESBD Diminished UVB-Induced Wrinkle Formation and Epidermal Thickness of Hairless Mouse Skin

**Figure 3:**
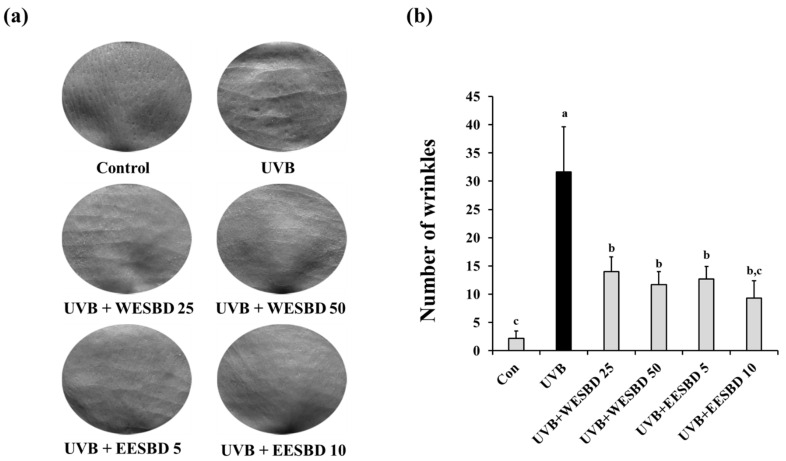
Ultraviolet B (UVB)-mediated wrinkle formation was attenuated by topical administration of water extract of *S. barbata* D. Don (WESBD) or ethanol extract of SBD (EESBD). At the end of the experiments, the number of wrinkles was calculated by a replica assay. Representative pictures of skin replica of each mouse group (*n* = 7) are shown (**a**). The number of wrinkles was counted under a microscope (**b**). Data are presented as the mean ± SD. Different letters indicate a significant difference between groups (*p* < 0.05).

**Figure 4:**
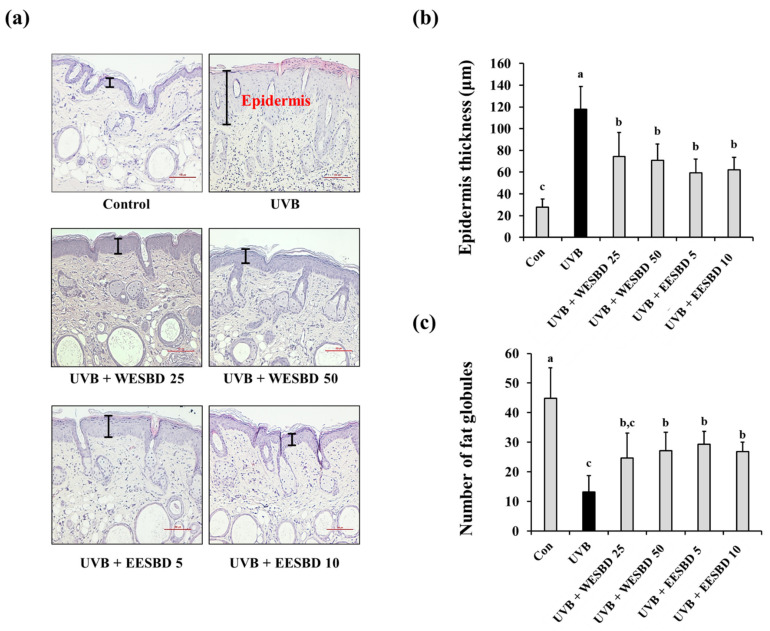
Effect of topical administration of water extract of *S. barbata* D. Don (WESBD) and ethanol extract of SBD (EESBD) on the epidermal thickness of hairless UVB-irradiated murine skin. Dorsal skin tissues (*n* = 7) were separated and fixed with 4% (*w*/*v*) paraformaldehyde solution, and the skin tissues were then embedded in paraffin and sliced into sections 5 μm thick and stained with hematoxylin and eosin (H&E) (**a**). The thickness of the epidermis (**b**) and number of globules in the dermis (**c**) in each mouse group were calculated under a microscope and tabulated. Data are presented as the mean ± SD. Different letters indicate a significant difference between groups (*p* < 0.05).

To further investigate the antiphotoaging activities of WESBD and EESBD, we examined their effects on UVB-induced changes in wrinkles and epidermal thickness of hairless mice. As shown in Figure 3, the number of skin wrinkles in the UVB group was markedly increased as compared with that in the control group. In contrast, the number of skin wrinkles in the WESBD- and EESBD-treated groups was significantly decreased as compared with that in the UVB group. Moreover, the epidermal thickness in the UVB group was markedly increased as compared with that in the control group (*p* < 0.05). As compared with the UVB group, the epidermal thickness was markedly decreased in the WESBD- and EESBD-treated groups (*p* < 0.05) (Figure 4a,b). Furthermore, epidermal fat loss induced by UVB irradiation, which is an indicator of photoaging, was not evident in the WESBD- and EESBD-treated groups (Figure 4a,c). Collectively, these results suggest that both WESBD and EESBD have antiphotoaging effects by ameliorating photoaging processes, such as wrinkle formation, epidermal thickening, and epidermal fat loss, induced by UVB.

### 2.4. Topical Administration of WESBD and EESBD Inhibited UVB-Induced Loss of Skin Collagen Content

**Figure 5:**
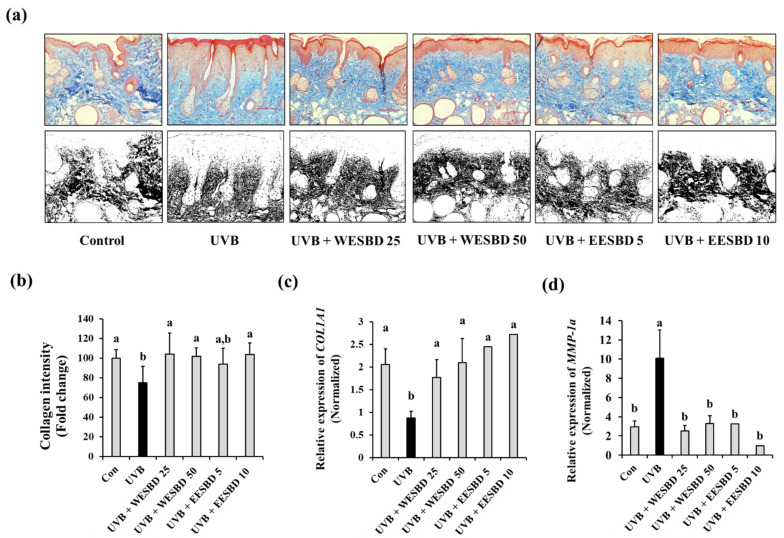
The change in the collagen contents of ultraviolet B (UVB)-irradiated mouse skin was recovered by topical administration of water extract of *S. barbata* D. Don (WESBD) or ethanol extract of SBD (EESBD). The tissue collagen content was visualized by Masson’s trichrome staining (**a**), upper panel) (*n* = 7). Only collagen-positive signals (blue) were converted to back color (**a**, lower panel) and the intensity of each black signal was calculated by using the ImageJ program and tabulated (**b**). The expression of type-1 procollagen (*COL1A1*) (**c**) and metalloproteinase-1a (*MMP-1a*) (**d**) in each mouse group (*n* = 7) was determined using quantitative real-time PCR (qRT-PCR) and normalized to that of the glyceraldehyde 3-phosphate dehydrogenase (*GAPDH*) gene. Data are presented as the mean ± SD. Different letters indicate a significant difference between groups (*p* < 0.05).

As shown in Figure 2, the WESBD and EESBD treatments restored the expression of *COL1A1* and *MMP-1a* in NIH-3T3 skin fibroblast cells (Figure 2). We confirmed the effects of both WESBD and EESBD on the collagen content of UVB-irradiated murine skin. As shown in Figure 5a,b, the dermal collagen content was significantly decreased in the UVB group as compared with that in the control group (*p* < 0.05). In contrast, as shown Masson’s trichrome staining, the collagen content was restored in the WESBD- and EESBD-treated groups (*p* < 0.05). In addition, *COL1A1* and *MMP-1a* expression were restored in the skin of the WESBD- and EESBD-treated groups (*p* < 0.05) (Figure 5c,d). These data strongly suggest that restoration of the collagen content of UVB-irradiated murine skin by WESBD or EESBD could be via the treatments’ effects on the expression of *COL1A1* and *MMP-1a*, both of which are closely linked to skin collagen synthesis and degradation.

### 2.5. WESBD and EESBD Attenuated UVB-Induced Abnormal Phosphorylation of AKT

**Figure 6:**
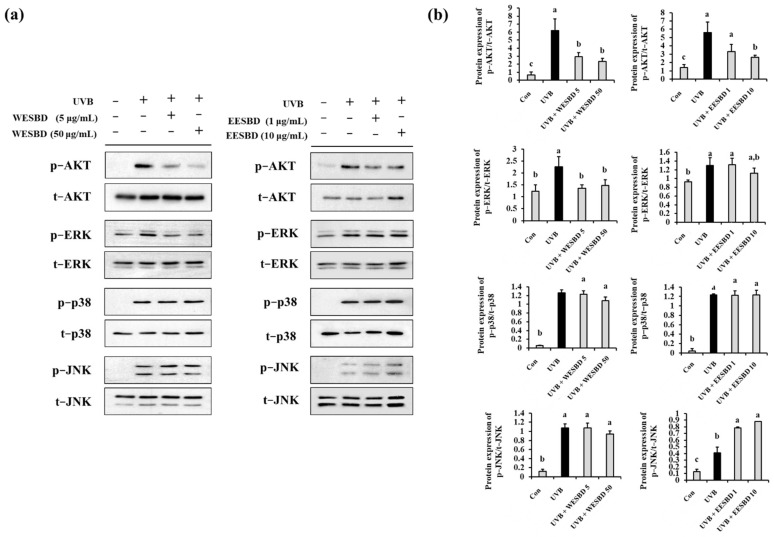
Ultraviolet B (UVB)-induced over-phosphorylation of protein kinase B (AKT) was suppressed by pretreatment with water extract of *S. barbata* D. Don (WESBD) or ethanol extract of SBD (EESBD). NIH-3T3 cells were pretreated with WESBD or EESBD, and the effects of UVB irradiation on AKT and mitogen-activated protein kinase (MAPK) phosphorylation were determined by Western blotting (**a**). The density of each band was calculated using the ImageJ program and tabulated (**b**). The experiments were repeated three times, with similar results. A representative result is shown. Data are presented as the mean ± SD. Different letters indicate a significant difference between groups (*p* < 0.05).

**Figure 7:**
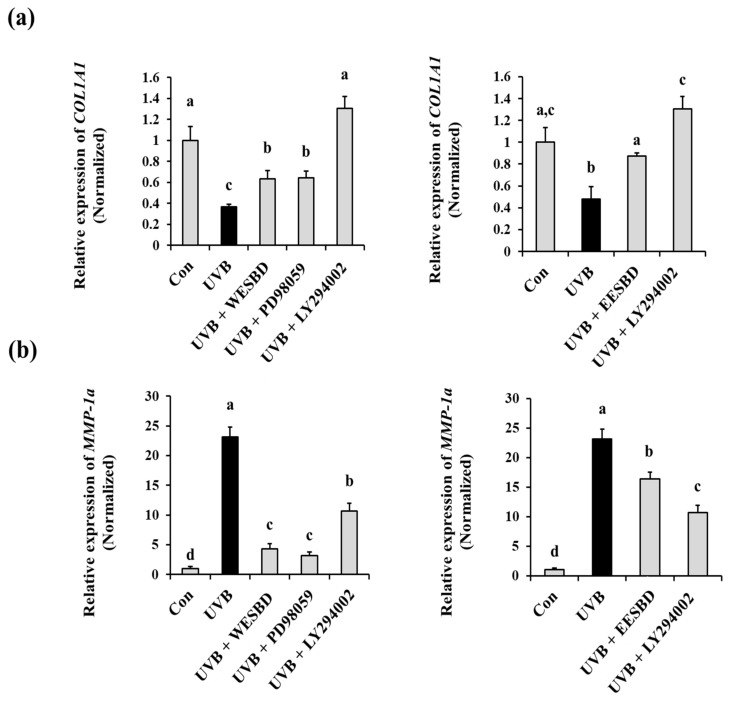
Comparison the anti-photoaging efficacy of water extract of *S. barbata* D. Don (WESBD) and ethanol extract of *S. barbata* D. Don (EESBD) with that of ERK and AKT inhibitors. The NIH-3T3 cells were pretreated with water extract of *S. barbata* D. Don (WESBD) (50 µg/mL), ethanol extract of SBD (EESBD) (10 µg/mL), an MEK inhibitor (PD98059, 10 µg/mL), and an AKT inhibitor (LY294002, 10 µg/mL), and the effects of UVB irradiation on *COL1A1* (**a**) and *MMP-1a* (**b**) were determined by qRT-PCR. The experiments were repeated three times, with similar results. A representative result is shown. Data are presented as the mean ± SD. Different letters indicate a significant difference between groups (*p* < 0.05).

To investigate the intracellular regulator that controls the antiphotoaging properties of WESBD and EESBD, we examined the effects of WESBD and EESBD on UVB-induced phosphorylation of AKT and MAPKs in NIH 3T3 cells by Western blotting. As presented in Figure 6a,b, the WESBD and EESBD treatments significantly reduced abnormal phosphorylation of AKT induced by UVB (*p* < 0.05). Although the WESBD treatment attenuated UVB-induced over-phosphorylation of ERK in the NIH 3T3 cells, it did not reduce abnormal phosphorylation of other MAPKS, such as p38 and JN, induced by UVB irradiation. In addition, the EESBD treatment did not inhibit abnormal phosphorylation of ERK, p38, and JNK induced by UVB irradiation. To shed further light on the efficacy of both WESBD and EESBD in inhibiting UVB-mediated photoaging processes, we compared *COL1A1* and *MMP-1a* expression in groups treated with different concentrations of WESBD and EESBD and ERK and AKT inhibitors. As depicted in Figure 7, the ability of WESBD at a concentration of 50 µg/mL to restore normal *COL1A1* and *MMP-1a* expression in the UVB-irradiated group was equivalent to that of the ERK inhibitor (10 µg/mL, PD98059). However, non-toxicological levels of WESBD (50 µg/mL) and EESBD (10 µg/mL) were less effective than the AKT inhibitor (10 µg/mL, LY294002) in restoring normal *COL1A1* and *MMP-1a* expression in the UVB irradiated group. Taken together, these results suggest that the antiphotoaging activity of WESBD and EESBD in terms of combatting UVB exposure involves inhibition of phosphorylation of AKT.

### 2.6. Single Components of EESBD as Determined by HPLC/MASS Analysis

**Figure 8:**
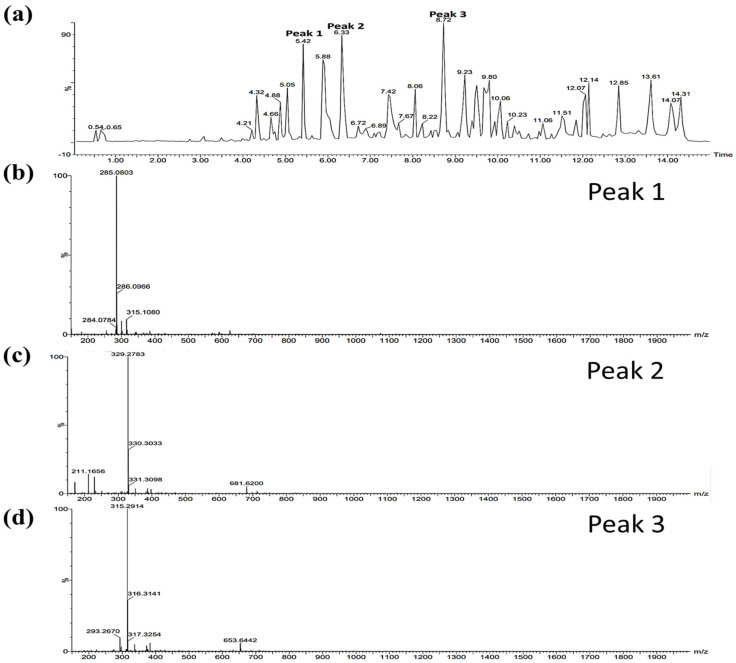
High performance liquid chromatography (HPLC) and mass spectrometry (MASS) analysis of ethanol extract of SBD (EESBD). A HPLC chromatogram of EESBD is shown (**a**). The major peaks (peak 1, peak 2, and peak 3) of the EESBD chromatogram were selected and further analyzed by MASS. The molecular weights of the three major peaks were estimated as 315.08 (**b**), 681.62 (**c**), and 653.64 (**d**) respectively.

The antiphotoaging activities of ESBD and EESBD were not significantly different in the various treatment groups. However, in terms of antiphotoaging effectiveness, EESBD (5–10 µg/mL) was more effective than WESBD (25–50 µg/mL) at a lower concentration (Figure 4 and Figure 5). Thus, EESBD was considered to have greater potential as an antiphotoaging agent, and we identified single components of EESBD by HPLC/MASS. Figure 8A shows the HPLC results for EESBD. Three major peaks (tR 5.42, tR 6.33, and tR 8.72) were analyzed using a mass spectrometer (Figure 8B,C,D). The observed masses of these three major peaks are listed in Table 1. Based on the results and previous literature, peaks 1 and 2 were tentatively deduced as isorhamnetin and scutebarbatine I, respectively. Although we did not elucidate the antiphotoaging properties of these three major components of EESBD in this study, the findings indicate that they could be used as analytical indicators of EESBD.

## 3. Discussion

Chronic UVB exposure is considered the main cause of photoaging processes, such as wrinkle formation and epidermal thickening. Excessive UVB exposure can induce cellular death, inflammation, and skin cancer [18,19]. Furthermore, UV rays are recognized as a negative regulator of subcutaneous fat synthesis, which plays an important role in endocrine and metabolic functions [3,20,21]. Therefore, it is important to develop protective agents that can inhibit the harmful effects of UVB rays and effectively protect skin from UVB-induced photoaging.

In this study, we evaluated the antiphotoaging efficacy of water and ethanol extracts of SBD (WESBD and EESBD, respectively). We found that non-toxicological levels of WESBD and EESBD effectively attenuated UVB-mediated wrinkle formation, epidermal thickening, and loss of epidermal fat, suggesting that both WESBD and EESBD have potential as natural antiphotoaging agents. In this study, both the WESBD and EESBD treatments restored UVB-mediated loss of epidermal collagen through recovering the UVB-induced abnormal expression of *COL1A1* and *MMP-1a*, which plays in role in maintaining the epidermal collagen content suggesting that both agents could be developed as potential antiphotoaging materials.

Many molecular mechanisms underlying antiphotoaging processes have been reported. Among these, MAPK and AKT signaling pathways have been well characterized and are known to be the main regulatory intracellular pathways involved in photoaging processes. Interestingly, many agents with antiphotoaging activity have similar effects on gene expression (e.g., *COL1A1* and *MMPs*), but these effects are exerted via different intracellular signaling molecules. For example, *Foeniculum vulgare* Mill extracts [6], ginseng protein [22], and vicenin-2 [11] were reported to exert antiphotoaging activities by inhibiting the phosphorylation of MAPKs (ERK, p38, and JNK). Safflower seed oil was reported to exert antiphotoaging activity by attenuating UVB-induced JNK phosphorylation but not ERK, p38, and AKT phosphorylation [23]. In natural agents, such as propolis [24] and brown pine leaf extracts [25], AKT but not MAPKs were reported to control the expression of antiphotoaging-related genes. Taken together, these reports suggest that although the antiphotoaging effects of many agents are similar, they utilize different intracellular signaling pathways to regulate the expression of genes associated with photoaging. In our study, although the ERK signaling pathway was involved in WESBD-mediated antiphotoaging, other MAPK molecules, such as p38 and JNK, were not involved in WESBD- and EESBD-mediated antiphotoaging process. In addition, UVB-induced AKT phosphorylation was significantly attenuated in the WESBD- and EESBD-treated NIH-3T3 cells. These findings indicate that AKT may be the main intracellular signaling molecule by which both WESBD and EESBD mediate antiphotoaging processes.

When developing functional agents from natural sources, such as plants and foods, determining the appropriate solvent for extraction is important to ensure functional materials that are economical and can be applied to the human body. For these reasons, we developed two different SBD-based antiphotoaging agents using ethanol or water as solvent for WESBD and EESBD, respectively. The cytotoxicity of WESBD and EESBD differed. In this study, we used the maximum doses of non-toxicological levels of EESBD and WESBD. Based on our findings, non-toxicological levels of both WESBD and EESBD showed similar levels of antiphotoaging efficacy. However, EESBD showed similar efficacy to WESBD in attenuating UVB-induced photoaging processes at a lower concentration. Thus, EESBD may be more advantageous than WESBD as an antiphotoaging agent in terms of industrial production. We also analyzed the single constituents of EESBD. Our results revealed that isorhamnetin and scutebarbatine I are the major single components of EESBD. The antiphotoaging activities of these single components will be investigated in a future study.

SBD is well-known herb used in traditional medicine with a long history as a heat-clearing and detoxifying agent. In terms of ethnopharmacological uses, SBD is widely used to suppress inflammation caused by traumatic injuries or snake bites. Recent pharmacological studies revealed that SBD has strong antibacterial activity [26] and that it may provide protection against cognitive deficits [27]. However, to the best of our knowledge, this study is the first to report antiphotoaging effects of SBD. The antiphotoaging effects of WESBD and EESBD observed in this study on UVB-irradiated NIH-3T3 cells and the hairless mouse model suggest that both WESBD and EESBD have potential as antiphotoaging agents.

## 4. Materials and Methods

### 4.1. Materials

Rabbit antiphospho-AKT (4060S), rabbit anti-AKT (9272S), rabbit antiphospho- extracellular signal-regulated kinase (ERK) (9102S), rabbit anti-ERK (9101S), rabbit antiphospho-c-Jun N terminal kinase (JNK) (9251S), rabbit anti-JNK (9252S), rabbit antiphospho-p38 (9212S), rabbit anti-p38 (9211S), MEK inhibitor (PD98059), and AKT inhibitor (LY294002) were obtained from Cell Signaling Technology, Inc. (Danvers, MA, USA).

### 4.2. Preparation of WESBD and EESBD

WESBD and EESBD were obtained from the Korea Plant Extract Bank (KPEB, Daejon, Korea) where voucher specimens are deposited (reference number: CW02-020 and CA01-061, respectively). SBD was authenticated by a botanist of KPEB, and the plant (100 g) dried in the shade until the moisture contents were reached to 5–10% according to the Korean Pharmacopoeia (KP) [28]. The dried SBD, which is stored for more than one year, was not used for the extraction. The dried SDB was ground into a powder and then added to 1 L of distilled water, followed by heat extraction for 150 min at 100 °C in an extractor (DW-290, Daewoong Electronic Appliance, Seoul, Korea). After filtration and freeze drying (Clean Vac 8; Hanil Science Inc, Gimpo, Korea), WESBD (11.7 g) was obtained. To obtain EESBD, SBD (62 g) was dried, powdered, and added to 1 L of ethyl alcohol 95.0% and extracted at room temperature using an ultrasonic extractor (SDN-900H; SD-Ultrasonic Co., Ltd., Seoul, Korea). After filtration (Hyundai Micro Co., Ltd., Seoul, Korea) and drying under reduced pressure, EESBD (4.2 g) was obtained.

### 4.3. Cytotoxicity Assay of WESBD and EESBD

NIH-3T3 skin fibroblast cells were purchased from the Korean Type Culture Collection (KTCC, Seoul, Korea). The cells were cultured in Dulbecco’s Modified Eagle’s Medium (DMEM) (HyClone, Logan, UT, USA) containing 10% (*w*/*v*) fetal bovine serum (Coring Company, Corning, NY, USA), 100 units/mL of penicillin, and 100 μg/mL of streptomycin. To assay the cytotoxicity of WESBD and EESBD, 1 × 10^4^ NIH-3T3 cells were seeded in each well of a 96-well flat-bottomed plate and incubated for 24 h at 37 °C in a CO_2_ incubator. The culture medium was then replaced with serum-free DMEM containing various concentrations of WESBD or EESBD (range: 0−300 μg/mL). After incubation for another 24 h, cell viability was estimated using WST-1 solution (Dozen, Seoul, Korea) according to the manufacturer’s protocols. The absorbance was evaluated at 450 nm using a microplate reader (iMark Microplate Reader; Bio-Rad Laboratories, Inc., Hercules, CA, USA).

### 4.4. UVB Treatment

DMEM containing WESBD or EESBD was preincubated with the NIH-3T3 cells for 24 h at 37 °C in a CO_2_ incubator, and the medium was then replaced with phosphate buffered saline (PBS) (1 mL). Subsequently, each well was irradiated with UVB (25 mJ/cm^2^) using a UV irradiation system (Bio-Link 312; Vilber Co., Suebia, Germany). These UVB-irradiated NIH-3T3 cells were then incubated with complete culture medium containing fetal bovine serum (10%), streptomycin (100 μg/mL), and penicillin (100 units/mL) for 24 h at 37 °C in a CO_2_ incubator.

### 4.5. Animal Study

Six-week-old female hairless mice (SKH-1; Orientbio Inc., Seoul, Korea) were used to study the antiphotoaging effects of WESBD and EESBD. After a 1-week adaptation period under controlled conditions (temperature: 23 ± 2 °C, humidity: 50 ± 10%, 12 h light/dark cycle), the mice were randomly divided into six groups (*n* = 7 per group): no treatment (control), UVB irradiation (UVB), UVB irradiation and pretreatment with WESBD 25 mg/kg/bw (UVB + WESBD 25), WESBD 50 mg/kg/bw (UVB + WESBD 50), EESBD 5 mg/kg/bw (UVB+ EESBD 5), and EESBD 10 mg/kg/bw (UVB+ EESBD 10). The SKH-1 hairless mice were treated with WESBD and EESBD dissolved in working solution (propylene glycol:ethanol = 7:3) and then exposed to UVB (50–200 mJ/cm; 1 minimal erythematous dose (MED): 50 mJ/cm^2^) every other day for 10 weeks using a microprocessor-controlled UV irradiation system (Bio-Link 312; Vilber Co., Suebia, Germany) The total amount of UVB irradiation was 78 MED (3900 mJ/cm^2^). The MED was defined as the dose of UVB radiation required to produce minimal erythema after 24 h.

### 4.6. Skin Replica Assay and Tissue Staining

At the end of experiment, to assess UVB-induced wrinkle formation, replicas were taken from the dorsal skin of each mouse using silicon impression material (Perfect-F Light Body Cartridge: Handae Chemical, Sungnam, Korea). The dorsal skin tissues were separated and fixed with 10% (*w*/*v*) paraformaldehyde solution. The skin tissues were then embedded in paraffin and sliced into sections 5 μm thick and stained with hematoxylin and eosin (H&E) and Masson’s trichrome solution for estimating changes in epidermal thickness and collagen fiber content, respectively.

### 4.7. Quantitative Real-Time PCR (qRT-PCR)

To determine the relative expression of photoaging-related genes, transcriptional expression was determined by qRT-PCR. Total RNA was isolated from the NIH-3T3 cells and murine skin using TRIzol^®^ reagent (Invitrogen, Waltham, MA, USA). Oligo dT primer (10 pM) and total RNA (1 µg) were used for the synthesis of complementary DNA. SYBR^®^ Green Realtime PCR Master Mix (Toyobo Co., Tokyo, Japan) and a RT-PCR detection system (CFX ConnectTM; Bio-Rad Co., Hercules, CA, USA) was used for qRT-PCR. The relative expression of each gene was estimated with the Ct method and normalized to that of glyceraldehyde 3-phosphate dehydrogenase (*GAPDH*). The primer sequences used in this study are listed in Table 2.

### 4.8. Western Blotting

To determine the effects of WESBD and EESBD on intracellular signaling pathways upregulated by UVB irradiation, the effects of the treatments on AKT and MAPK phosphorylation of the NIH-3T3 cells were evaluated by Western blotting. After cell lysis in lysis buffer [29] for 1 h. The cell lysates were centrifuged (13,000× *g*, 4 °C, 15 min). The protein concentration in the separated supernatant was measured using the Bradford assay [30]. For estimating the expression of each molecule, 20 µg of cell lysates were electrophoresed in sodium dodecyl sulfate polyacrylamide gel and then transferred to nitrocellulose membranes. After incubation with blocking buffer (5% *w*/*v* of nonfat milk in tris-buffered saline with Tween 20 (TBS-T) for 1 h), the membrane was reacted with a primary antibody (1:5000) for 16 h at 4 °C. After washing with TBS-T buffer three times, the membrane was reacted with a secondary antibody (1:3000) and conjugated with horseradish peroxidase (Santa Cruz, Dallas, TX, USA) at room temperature for 2 h. Each protein band was visualized using an enhanced chemiluminescence detection kit (Bio-Rad, Hercules, CA, USA).

### 4.9. Single Components Analysis

Single components in EESBD were identified using an AQUITY Ultra Performance LCTM system (Waters Corp., San Jose, CA, USA) coupled with a Micromass Q-Tof PremierTM mass spectrometer (Waters Corp, San Jose, CA, USA). EESBD was separated on a BEH C18 column (100 mm × 2.10 mm, 1.7 µm) (Thermo Fisher Scientific, San Jose, CA, USA) in two mobile phases: eluent A (aqueous formic acid solution, 0.1% *v*/*v*) and eluent B (acetonitrile with formic acid, 0.1%, *v*/*v*). The flow rate was 0.4 mL/minute at 40 °C. The Micromass Q-Tof PremierTM mass spectrometer (Waters Corp, San Jose, CA, USA) was operated in the negative mode from 100 to 2000 Da, with a 0.2 s scan time and desolvationary temperature of 350 °C; desolvation gas flow of 800 L/hour (N2), source temperature of 110 °C, cone voltage of 50 V, and capillary voltage of 2.3 kV.

### 4.10. Statistical Analysis

All data are presented as the mean ± standard deviation (SD). A two-tailed, unpaired Student’s *t*-test and an analysis of variance using Prism 5 software (Graph-Pad Software, Inc., San Diego, CA, USA) were used for statistical analysis. A value of *p* < 0.05 was considered statistically significant.

## Figures and Tables

**Table 1:** Major components of water extract of *S. barbata* D. Don.

Natural Product	Peak	RT (min)	Observed Mass	Fragmentation	Single Compound	Formula	Molecular Mass (g/mol)	Ref.
*S.barbata* D. Don	1	5.42	315.1080	285.0803	Isorhamnetin	C16H12O7	316.26	[14]
	2	6.33	681.6200	329.2783, 211.1656	Scutebarbatine I	C38H39N3O9	681.7	[14]
	3	8.72	653.6442	315.2914, 293.2670	Not determined	-	653.12	-

**Table 2:** Primer sequences.

Genes	Sequences	Species
*COL1A1*	5′- CACTGCTGTTGGTCCACGT -3′ (Forward) 5′- AAAGCACAGCACTCGCCC -3′ (Reverse)	Mouse
*MMP-1a*	5′- ACTTTCCAGCCAGGCCCA -3′ (Forward) 5′- CACTGCTGTTGGTCCACGT -3′ (Reverse)	Mouse
*IL-6*	5′- ACAACCACGGCCTTCCCT -3′ (Forward) 5′- AGCCTCCGACTTGTGAA -3′ (Reverse)	Mouse
*IL-8*	5′- TGTCCCATGCCACTCAGAGA -3′ (Forward) 5′- AGCAGGTGCTCCGGTTGTAT -3′ (Reverse)	Mouse
*MCP-3*	5′- ATAGCCGCTGCTTTCAGCAT -3′ (Forward) 5′- CTTCCCAGGGACACCGACTA -3′ (Reverse)	Mouse
*GAPDH*	5′- AAGCTGTGGCGTGATGGC -3′ (Forward) 5′- TGACCTTGCCCACAGCCT -3′ (Reverse)	Mouse

## Data Availability

Not applicable.

## Sample Availability

Samples of WESBD and EESBD may be available on request from corresponding authors.
